# Pandemic-Related Black Family Well-Being Across North Carolina County Tiers: An Exploratory Cross-Sectional Study

**DOI:** 10.3390/ijerph23070856

**Published:** 2026-06-30

**Authors:** Chima Okoli, Tony Bawo Esimaje, Nina Smith, Timothy J. Mulrooney, Fredrick Johnson, The National African American Child and Family Research Center

**Affiliations:** 1 Integrated Biosciences Doctoral Program, North Carolina Central University, Durham, NC 27707, USA; tesimaje@eagles.nccu.edu; 2 College of Health and Sciences, North Carolina Central University, Durham, NC 27707, USA; nsmith42@nccu.edu; 3 Department of Environmental, Earth and Geospatial Sciences, North Carolina Central University, Durham, NC 27707, USA; tmulroon@nccu.edu; 4 Department of Mathematics and Physics, North Carolina Central University, Durham, NC 27707, USA; fjohn-son@nccu.edu

**Keywords:** Black families, county tier, COVID-19, family well-being, health equity, North Carolina

## Abstract

**Highlights:**

**Public health relevance—How does this work relate to a public health issue?**

- COVID-19 disrupted multiple domains of Black family life, including emotional well-being, food access, sleep, work, and parenting.
- This study examines within-group variation across North Carolina county economic tiers, rather than treating Black families as a homogeneous population.

**Public health significance—Why is this work of significance to public health?**

- Most cross-tier comparisons were not statistically significant, indicating that reported burden was not neatly separated by county tier.
- The county-tier system provides policy-relevant context but is too coarse to explain family level well-being on its own.

**Public health implications—What are the key implications or messages for practitioners, policy makers, and researchers?**

- Public health surveillance should pair county-tier classifications with neighborhood, household, service-access, and social-support measures.
- Larger longitudinal studies are needed before tier-specific interventions or causal explanations are inferred.

**Abstract:**

This exploratory cross-sectional study examined whether pandemic-related family well-being responses differed across North Carolina’s 2021 county economic tiers among 178 Black parents. Survey responses were linked to county tier and included reported stress, emotional symptoms, food hardship, self-rated health, sleep change, work and parenting disruption, and parent–child interaction items. Primary analyses used the Kruskal–Wallis and chi-square tests; within-tier correlations and tier-stratified linear probability models were used for supplementary and descriptive purposes. Respondents across all tiers reported pandemic-related burdens, but most cross-tier comparisons were not statistically significant. One food-hardship item (*p* = 0.050) and one parent–child interaction item (*p* = 0.042) met the nominal 0.05 threshold, while one emotional symptom item approached significance (*p* = 0.074); these isolated findings did not form a consistent cross-domain pattern. The findings indicate that the county-tier classification was useful for organizing place-based comparisons but did not consistently differentiate response patterns in this sample. Larger, longitudinal studies using neighborhood- and household-level measures are needed before tier-specific or causal conclusions can be drawn.

## 1. Introduction

The COVID-19 pandemic intensified longstanding health and socioeconomic inequities in the United States. In a systematic review of early U.S. evidence, studies detecting disparities estimated 1.5–3.5 times higher SARS-CoV-2 infection risk and 1.5–3 times higher hospitalization risk for Black than White populations [1]. Black communities also experienced concentrated exposure to structural conditions affecting employment, material security, caregiving, and access to health-promoting resources [2,3]. Although much pandemic research has compared racial and ethnic groups, less is known about variations among Black families across local contexts. This within-group question matters because state and local agencies often allocate resources geographically, while racial classification alone does not indicate which local constraints or supports families encounter.

Families with children experienced overlapping disruptions during the pandemic, including job and income instability, food hardship, childcare changes, sleep disruption, emotional distress, and increased caregiving demands [4,5,6]. These disruptions can affect both individual well-being and family processes. Family stress perspectives indicate that economic and health-related pressures may spill over into caregiver emotional availability, household routines, and parent–child interactions. Families may nevertheless respond differently depending on employment flexibility, household resources, social support, and the services available in their communities.

North Carolina’s county-tier system provides one policy-relevant way to organize local economic context. For the 2021 designations used in this study, the North Carolina Department of Commerce ranked all 100 counties using four factors: the 12-month average unemployment rate, median household income, 36-month population growth, and adjusted property tax base per capita. The factor ranks were combined to order counties by economic distress. Tier 1 represented the most economically distressed counties, Tier 2 represented intermediate distress, and Tier 3 represented the least distress; a tie in the 2021 ranking resulted in 41 Tier 1 counties, 39 Tier 2 counties, and 20 Tier 3 counties [7]. The classification is used in state economic-development programs, but it is an aggregate county measure rather than a direct measure of household socioeconomic position, structural racism, neighborhood conditions, or access to services.

Structural racism in housing, labor markets, education, and public investment has contributed to unequal access to economic opportunity and stabilizing resources [8]. Ecosocial theory similarly emphasizes how multilevel social conditions become embodied through repeated exposure to risk and constraint [9]. Within-group heterogeneity is therefore analytically important: Black families may share exposure to racialized structural disadvantage while differing in household resources, extended-family support, and local opportunity structures [10]. Relevant pandemic pathways include precarious employment and work–family conflict [11,12,13,14], family stress and resilience processes [15,16], food insecurity and parental strain [17,18], and heightened stress, anxiety, and depressive symptoms among Black adults and caregivers [3,19,20]. Together, this literature supports examining emotional symptoms, food hardship, work and parenting disruption, sleep, health, and parent-child interaction without assuming that a single county classification fully captures these experiences.

### Study Purpose and Research Questions

The purpose of this study was to assess pandemic-related family well-being among Black parents across North Carolina’s county-tier classification. The analysis was guided by three research questions:

1. RQ1. Do Black parents’ pandemic-related emotional symptoms, food-hardship responses, and parent–child interaction responses differ across North Carolina county tiers?
2. RQ2. Within each tier, how are selected work, parenting, sleep, health, and material-hardship indicators descriptively associated with emotional symptoms and food hardship?
3. RQ3. Do selected work, parenting, health, sleep, and material-hardship indicators show different supplementary association patterns with reported stress within county tiers?

## 2. Materials and Methods

### 2.1. Study Design and Context

This study used a cross-sectional electronic survey to examine family well-being among Black parents living in North Carolina during the COVID-19 pandemic. The questionnaire assessed demographic characteristics, employment disruption, food hardship, self-rated health, sleep change, reported stress, emotional symptoms, and parent–child interaction patterns. Each participant’s self-reported county of residence was linked to the 2021 North Carolina county-tier designation. County tier was used as a broad contextual grouping variable for exploratory comparison, not as a precise or exhaustive measure of structural exposure.

### 2.2. Sampling Strategy and Participants

Eligibility criteria included (1) self-identification as Black or African American, (2) age of 18 years or older, and (3) being the parent or guardian of at least one minor child. Participants were recruited through statewide urban and rural outreach to complete an electronic survey. Recruitment was nonprobability-based. The historical recruitment documentation did not retain a channel-by-channel breakdown of recruitment sources, so recruitment source effects could not be evaluated.

After screening, obtaining consent, and linking to county data, the analytic sample included 178 respondents: Tier 1 (n = 34), Tier 2 (n = 92), and Tier 3 (n = 52). These uneven subgroup sizes supported exploratory descriptive and nonparametric analyses but reduced statistical power and precision, particularly for tier-specific multivariable models.

### 2.3. Measures

#### 2.3.1. Demographic and Household Characteristics

The questionnaire assessed gender, age, education, household income, marital or relationship status, number of children, number of adults in the household, number of children in the household, and child age and gender. These items were used to characterize the study sample and confirm eligibility.

#### 2.3.2. Pandemic-Related Work, Parenting, Health, Sleep, and Material-Hardship Indicators

Employment and work disruption were assessed with items asking whether the respondent was employed before COVID-19, the respondent’s current employment status during COVID-19, whether the respondent lost a job because of COVID-19, and whether COVID-19 affected the respondent’s ability to work. The analysis used binary indicators for job loss due to COVID-19 and work impact. Material support was assessed through items asking whether anyone in the household received food stamps and whether benefits had been received before COVID-19. Sleep was assessed with a five-category item asking whether sleep quality had changed since the onset of COVID-19. Self-rated health was assessed using a four-category item ranging from excellent to poor; self-rated health has established predictive validity in population research [21].

Parenting disruption was measured with a binary item asking whether COVID-19 affected parenting practices. Reported pandemic-related stress was measured with a binary gateway item asking whether the respondent had been stressed since the onset of COVID-19. This binary stress item was used as a broad descriptive indicator and as the outcome in the supplementary tier-stratified association models.

#### 2.3.3. Perceived Stress and Emotional Symptoms

Perceived stress was assessed using 10 Likert-style items asking how often respondents had experienced specific stress-related experiences in the previous few weeks, including feeling upset by unexpected events, unable to control important things, nervous or stressed, unable to cope, angered by events outside one’s control, and overwhelmed by accumulating difficulties [22]. Positively worded items included confidence in handling personal problems, feeling that things were going one’s way, controlling irritations, and feeling on top of things.

Emotional symptoms were assessed using 10 items asking how often respondents had experienced specific emotional states since the onset of COVID-19. The item set reflected commonly assessed depressive-symptom content, including concentration difficulty, depressed mood, effort, hopefulness, fear, restless sleep, happiness, loneliness, and difficulty getting going [23]. The items were analyzed conservatively as exploratory indicators rather than presented as a clinical diagnosis or a complete standardized scale.

#### 2.3.4. Food Hardship

Household food hardship was assessed using 10 items adapted from the U.S. household food security survey tradition [24]. Items covered worry that food would run out, food not lasting, inability to afford balanced meals, inability to feed children balanced meals, cutting or skipping meals, eating less than needed, hunger due to lack of money for food, not eating for a whole day, cutting children’s meal sizes, and children being hungry because the household could not afford more food. Responses were coded as never true, sometimes true, or often true.

#### 2.3.5. Parent–Child Interaction

Parent–child interaction was assessed using a 16-item module that asked respondents to select one child and indicate whether each interaction statement applied to the mother only, the father only, both the mother and father, or neither. Items included losing one’s temper, having fun together, taking frustration out on the child, not really listening, yelling, laughing often, threatening punishment, praising the child, being together but not really interacting, being too tired to interact, warmth, disapproval, togetherness, punishment, affection, and not paying much attention.

Because the response options identified caregiver configuration rather than frequency, these items were treated as categorical distributions and analyzed with chi-square tests rather than converted into continuous parenting scores.

### 2.4. Questionnaire Domains and Analytic Use

Table 1 summarizes how the questionnaire domains were used in the analysis. The table makes the connection between the survey instrument and the analytic plan explicit.

### 2.5. Data Processing

Data processing proceeded in several steps. First, records were screened for consent, race, age, and parent or guardian eligibility. Second, county of residence was mapped to the 2021 county-tier designation, and records without a valid county linkage were excluded from tier-stratified analyses. Binary indicators, such as work impact and job loss, were coded as 0/1. Ordinal measures were retained in their ordered form for descriptive and nonparametric comparisons; simplified binary versions were used only when required for the supplementary models. Summary indicators were developed to describe stress and food-hardship domains, while item-level tests were treated as exploratory follow-up assessments rather than independent confirmatory tests. Available-case analysis was used for descriptive summaries and index construction, and listwise deletion was used for the supplementary regression models. These choices were made for transparency but can reduce precision when data are missing [25].

### 2.6. Statistical Analysis

The analysis was designed to identify descriptive cross-tier patterns, not to estimate causal effects. County tier was the main grouping variable. Because many outcomes were ordinal, categorical, or non-normally distributed, primary cross-tier analyses used nonparametric and categorical tests.

Kruskal–Wallis tests were used for ordinal or item-level emotional symptom and food-hardship indicators [26]. Chi-square tests were used for parent–child interaction items because the response categories identified whether each statement applied to the mother only, father only, both parents, or neither. These cross-tier tests constituted the primary analytic evidence.

Spearman correlations were used as secondary exploratory analyses to describe within-tier associations among stress-related indicators, food hardship, sleep change, work disruption, parenting disruption, job loss, SNAP receipt, and self-rated health [27]. Finally, tier-stratified linear probability models were included as supplementary descriptive analyses, using the binary reported-stress item as the outcome. Predictors were work impact, parenting impact, sleep change, self-rated health, food-stamp receipt, and job loss due to COVID-19. The linear probability specification was used for transparent interpretation of associations with a binary outcome, with results treated as exploratory rather than definitive [28].

Because the analyses included several related item-level comparisons, isolated *p*-values at or near 0.05 were treated as possible signals rather than as confirmatory evidence of tier-based differences [29]. Interpretation emphasized consistency across domains and the precision of estimates rather than individual *p*-values alone.

### 2.7. Heatmap Visualization of Sample Distribution

A heat map was created in ArcGIS Pro (Version 3.4) to show the geographic clustering of respondents across North Carolina counties and was overlaid on the 2021 economic tiers. The visualization provides descriptive context for the sample distribution; it was not used to test spatial effects or infer county-level prevalence.

### 2.8. Ethical Considerations

The study was approved by the Institutional Review Board of North Carolina Central University. All participants provided informed electronic consent before participation. Confidentiality was maintained through de-identification and secure data storage. The consent materials described minimal risks, voluntary participation, compensation conditions, and contact information for compliance inquiries.

## 3. Results

### 3.1. Sample and County-Tier Distribution

**Figure 1:**
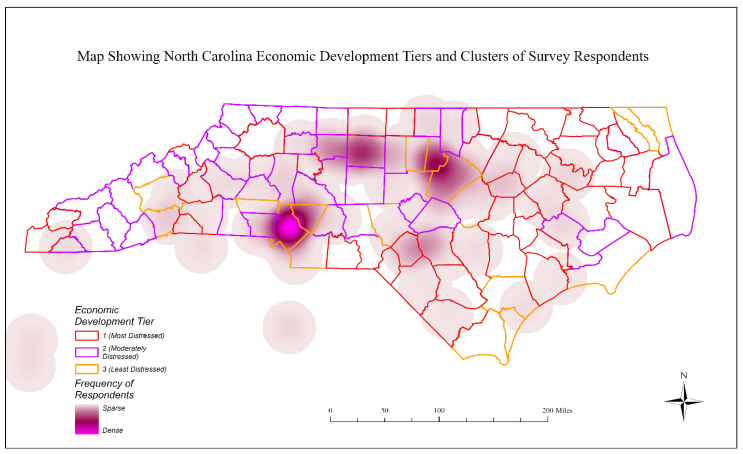
Heatmap showing the distribution of survey respondents across North Carolina counties and 2021 economic development tiers.

The analytic sample included 178 Black parents in North Carolina: Tier 1 (n = 34), Tier 2 (n = 92), and Tier 3 (n = 52). Figure 1 shows the geographic clustering of respondents across North Carolina counties, overlaid on the county’s economic tiers.

Responses were concentrated in the central and southern parts of the state, although all three tiers were represented. The concentration may reflect both recruitment reach and participation patterns; because county-level recruitment-source data were not retained, the contribution of recruitment strategy to the observed clustering cannot be quantified. The map should therefore be interpreted as providing sample context rather than as evidence of spatial effects.

### 3.2. Primary Cross-Tier Findings

The primary cross-tier analyses did not show strong or consistent evidence of systematic tier-based separation in pandemic-related family well-being. Across emotional symptoms, food-hardship indicators, and parent–child interaction items, most comparisons were not statistically significant. A small number of item-level differences or near-differences emerged, but these findings were isolated and did not form a consistent pattern across domains.

At least some respondents in every tier reported stress, emotional symptoms, food hardship, and parenting-related disruption. However, the cross-tier tests evaluated differences in response distributions; they did not estimate the pandemic’s causal effect, population prevalence, or change from a pre-pandemic benchmark. The results therefore support a conclusion of reported burden across all tiers, not a claim that one tier experienced systematically greater burden.

### 3.3. Emotional Symptom Patterns Across Tiers

Emotional distress was reported across all county tiers. Kruskal–Wallis tests for emotional symptom items did not show a consistent pattern of statistically significant tier differences as shown in Table 2 below. One item, trouble keeping one’s mind on what one was doing, approached statistical significance (H = 5.20, *p* = 0.074). This result suggested possible variation in concentration difficulty across tiers, but it did not support a substantive claim of tier-based separation in emotional symptoms.

**Figure 2:**
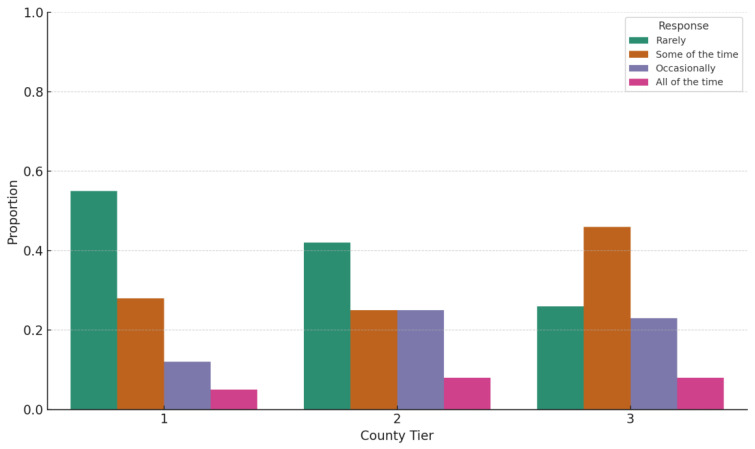
Normalized proportional responses for the emotional symptom item ‘Had trouble keeping my mind on what I was doing’ by county tier.

Figure 2 displays tier-specific response proportions for the concentration-difficulty item, which had the smallest *p*-value among the emotional symptom comparisons. Although the distributions varied descriptively, the broader emotional symptom domain remained largely similar across tiers.

### 3.4. Food-Hardship Patterns Across Tiers

Food-hardship indicators also showed limited cross-tier separation. Across the 10 item-level comparisons, one child-hunger item reached the nominal threshold of statistical significance (*p* = 0.050) as shown in Table 3 below. Given the number of item-level tests and the modest subgroup sizes, this result should be interpreted cautiously.

**Figure 3:**
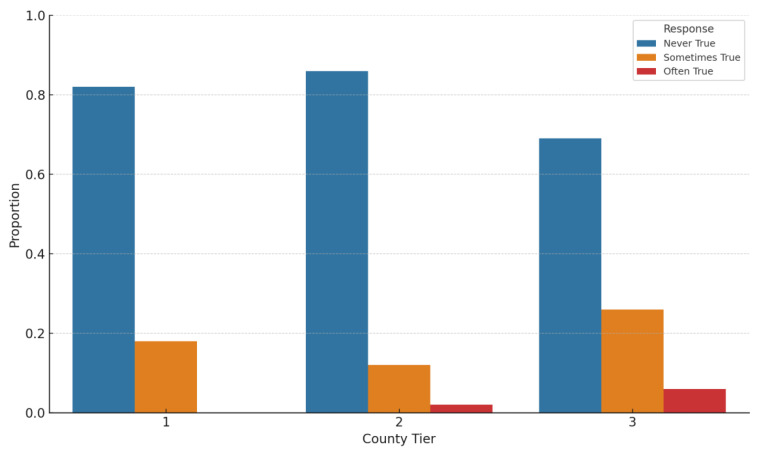
Normalized proportional responses for the food-hardship item ‘My/our children were hungry because we could not afford more food’ by county tier.

Figure 3 displays the normalized proportional responses for the child-hunger item. The descriptive distributions indicate that the item was endorsed in each tier, while the isolated *p*-value does not establish a general tier pattern.

### 3.5. Parent–Child Interaction Patterns Across Tiers

Parent–child interaction items showed limited evidence of systematic cross-tier differentiation. Because the response options identified whether each statement applied to the mother only, the father only, both parents, or neither, the items were analyzed as categorical distributions. Table 4 summarizes the findings. Of the 16 items examined, only ‘Didn’t really listen to what your child had to say’ showed a statistically significant chi-square result (chi-square = 13.08, *p* = 0.042). In the context of multiple exploratory item-level comparisons, this result is best interpreted as a possible signal rather than definitive evidence of broad parenting differences by county tier.

**Figure 4:**
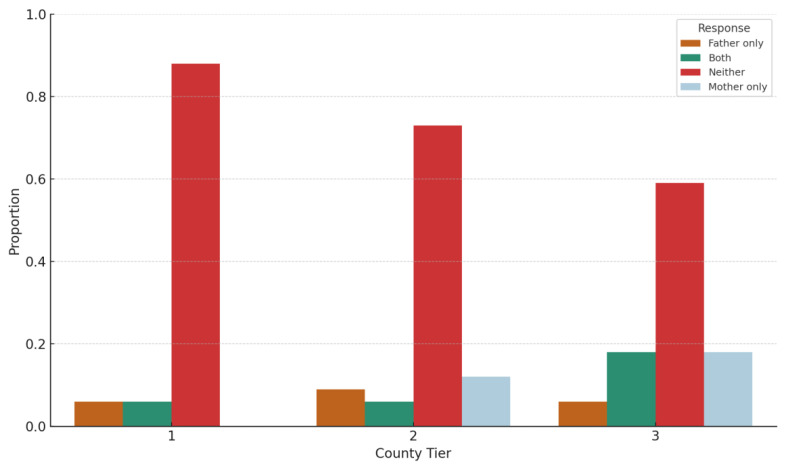
Normalized proportional responses for the parent–child interaction item ‘Didn’t really listen to what your child had to say’ by county tier.

Figure 4 presents the normalized proportional responses for this parent–child interaction item.

### 3.6. Exploratory Within-Tier Association Patterns

Secondary within-tier Spearman correlations were used to assess whether associations among emotional symptoms, food hardship, and selected pandemic-related disruptions varied across county tiers. These correlations were exploratory and hypothesis-generating and were not interpreted as causal effects.

For emotional symptoms, notable descriptive patterns included associations with poor sleep, self-rated health, and parenting disruption in Tier 1; work disruption and food-stamp receipt in Tier 2; and parenting disruption and sleep change in Tier 3. For food hardship, descriptive patterns suggested clustering with job loss and sleep disruption in Tier 1, work and parenting disruption in Tier 2, and self-rated health in Tier 3.

These within-tier associations suggest that burdens may have clustered with different household and health indicators across tiers, even though the primary cross-tier comparisons did not show strong systematic separation.

### 3.7. Supplementary Tier-Stratified Association Models of Reported Stress

Supplementary tier-stratified linear probability models were estimated to describe whether selected pandemic-related disruptions showed different association patterns with the binary reported-stress item within county tiers. These models followed the primary cross-tier analyses and were not the main evidentiary basis of the study. Table 5 below summarizes the findings.

In Tier 1, the model was not statistically significant (adjusted R^2^ = −0.129, *p* = 0.768), and no individual predictor reached statistical significance. These results did not identify a stable pattern of within-tier predictors for reported stress in the most economically distressed counties.

In Tier 2, the model was not statistically significant overall (adjusted R^2^ = 0.029, *p* = 0.226). The coefficient for work disruption was statistically significant, but the weak overall model makes this an isolated descriptive finding rather than a robust tier-specific effect.

In Tier 3, the model was statistically significant overall (adjusted R^2^ = 0.171, *p* = 0.033), but no individual predictor reached statistical significance. This pattern indicates a limited model-level signal without a clearly identifiable single driver of reported stress in the least distressed counties.

Taken together, the supplementary models did not support strong claims that a stable set of measured disruptions explained reported stress differently across tiers. They reinforce the conclusion that the measured household, work, health, and family process conditions were only partially represented by the available survey variables.

## 4. Discussion

The principal finding was that pandemic-related stress, emotional symptoms, food hardship, and parenting-related disruption were reported in every county-tier group, while most cross-tier comparisons were not statistically significant. Thus, the results indicate a broadly shared burden in this sample rather than a consistent separation between more- and less-distressed county tiers. This distinction is important: the study describes how responses were distributed across tiers, but it does not quantify a causal pandemic effect or population prevalence for all Black families in North Carolina.

The shared cross-tier pattern is consistent with research describing widespread disruptions to caregiver mental health and family functioning during COVID-19 [4,5,19,20]. Family stress models explain why work instability, material hardship, health concerns, and caregiving demands may affect emotional well-being, routines, and parent–child interaction, even when families live in different county economic contexts [15,30]. Prospective evidence also links maternal and child distress with family strain during the pandemic [31]. The present study did not test these mechanisms directly, but its domain-level pattern is compatible with the expectation that multiple stressors can converge within family systems.

The food-hardship findings also align with national evidence that food insecurity increased early in the pandemic and was unequally distributed across racial and household groups [17,18]. The single child-hunger item at *p* = 0.050 should not be interpreted as establishing a tier gradient. Instead, the presence of food-hardship responses in all three tiers supports examining household income, benefit access, transportation, school meal disruption, and local food availability directly in future work rather than relying on county tier as a substitute for those mechanisms.

The supplementary models provide similarly limited evidence of tier-specific processes. The isolated Tier 2 association between work disruption and reported stress is directionally consistent with research linking occupational and pandemic-related stress [14,32], but the overall Tier 2 model is not statistically significant. Likewise, the significant Tier 3 model did not identify a significant individual predictor. These results are appropriately treated as hypothesis-generating and do not establish that work or other disruptions operated differently by tier.

The limited cross-tier differentiation clarifies both the value and the limits of the state classification. County tier is useful because it is a transparent, policy-relevant grouping based on four county economic indicators [7]. It is coarse because the same tier can contain households and neighborhoods with very different incomes, employment conditions, transportation options, school and childcare resources, health services, and social-support networks. Structural and ecosocial perspectives therefore support pairing county-level classifications with more proximal measures rather than interpreting tier as a stand-alone explanation of family well-being [8,9,10].

The isolated emotional symptom, food-hardship, and parent–child interaction results should also be interpreted in the context of multiple related tests. One emotional item approached significance, one food-hardship item met the nominal 0.05 threshold, and one parent–child interaction item was statistically significant. Because these findings did not align into a consistent cross-domain pattern, the evidence does not support distinct tier-based well-being profiles. The more supportable interpretation is that burden was reported across tiers and that the county-tier measure did not consistently distinguish the response distributions.

### Limitations and Implications for Future Research

Several limitations constrain interpretation. The nonprobability, cross-sectional electronic survey design limited generalizability and temporal inference. Historical recruitment records did not retain a channel-level breakdown, so the extent to which the geographic concentration of responses reflected recruitment reach could not be quantified. Tier-specific subsamples were modest and uneven, especially in Tier 1, reducing precision and statistical power. Measures were self-reported, and some survey items were analyzed individually rather than as complete standardized scales. The county-tier designation is an aggregate contextual proxy and cannot capture within-county heterogeneity or more proximal determinants of family well-being. The supplementary regression models should be read only as descriptive sensitivity analyses.

Future research should use larger probability-based or carefully stratified samples, longitudinal designs, and contextual measures below the county level. A stronger design would retain county tier for policy relevance while adding household income and employment conditions, neighborhood deprivation, school and childcare access, transportation, health and mental health service availability, food access, and social support. Such designs would permit better-powered multilevel analyses and a more defensible assessment of whether local context modifies pandemic-related family outcomes.

## 5. Conclusions

Pandemic-related stress, emotional symptoms, food hardship, and parenting strain were reported among Black parents in all three North Carolina county-tier groups, while the tier classification did not consistently separate response patterns. The main public health takeaway is therefore not that one socioeconomic tier was uniquely affected, but that burden extended across the county economic spectrum represented in the sample. Because the tier designation is built from four county-level economic indicators, it can organize policy-relevant comparisons but cannot explain household- and neighborhood-level variation on its own. Future studies should combine this broad policy lens with finer contextual measures, larger samples, and longitudinal designs before drawing tier-specific or causal conclusions.

## Figures and Tables

**Table 1:** Questionnaire domains, item sources, and analytic roles.

Construct	Questionnaire Source	Analytic Role
County tier	County of residence mapped to the 2021 North Carolina county tier	Main grouping variable
Job loss due to COVID-19	Item asking whether the respondent lost a job because of COVID-19	Pandemic disruption indicator
Work impact	Item asking whether COVID-19 affected their ability to work	Pandemic disruption indicator
Parenting impact	Item asking whether COVID-19 affected parenting practices	Pandemic disruption indicator
Reported stress	Binary item asking whether the respondent had been stressed since COVID-19 began	Descriptive stress indicator and supplementary model outcome
Sleep change	Five-category item on whether sleep quality changed since COVID-19 began	Health and disruption indicator
Self-rated health	Four-category item ranging from excellent to poor	Health indicator
Food hardship	Ten household food-status items	Food-hardship domain
Parent–child interaction	Sixteen caregiver–child interaction items with mother/father/both/neither response categories	Categorical family process domain
Emotional symptoms	Ten symptom items since COVID-19 began	Emotional well-being domain
Perceived stress	Ten stress-frequency items during the previous few weeks	Stress domain

**Table 2:** Emotional symptom item-level cross-tier tests.

Emotional Item	Kruskal–Wallis H	*p*-Value
Had trouble keeping my mind on what I was doing	5.20	0.074
Felt depressed	1.82	0.403
Everything I did was an effort	1.56	0.457
Could not get going	0.99	0.608
Sleep was restless	0.73	0.694

**Table 3:** Food-hardship item-level cross-tier tests.

Food-Hardship Item	Kruskal–Wallis H	*p*-Value
Children were hungry	5.99	0.050 *
Could not afford balanced meals	4.27	0.118
Did not eat because could not afford food	4.23	0.121
Had to cut children’s meals	4.17	0.125
Ate less than we should	3.96	0.138

* Nominal *p* < 0.05. The item-level result is interpreted as exploratory because multiple related comparisons were conducted.

**Table 4:** Chi-square tests for parent–child interaction items.

Parent–Child Interaction Item	Chi-Square	df	*p*-Value
Didn’t really listen to what child had to say	13.08	6	0.042 *
Threatened to punish child	9.33	6	0.156
Was too tired to interact much	8.72	6	0.190
Was not paying much attention to child	7.66	6	0.264
Showed disapproval of something child did	6.04	6	0.419

* Nominal *p* < 0.05. The item-level result is interpreted as exploratory because multiple related comparisons were conducted.

**Table 5:** Supplementary tier-stratified regression summary.

Tier	N	R^2^	Adj. R^2^	Model *p*	95% CI: Work Impact	95% CI: Parenting Impact	95% CI: Job Loss
Tier 1	34	0.154	−0.129	0.768	[−0.22, 1.12]	[−0.48, 0.83]	[−0.13, 1.03]
Tier 2	92	0.101	0.029	0.226	[0.03, 0.49] *	[−0.11, 0.51]	[−0.24, 0.56]
Tier 3	52	0.279	0.171	0.033 *	[−0.04, 0.58]	[−0.12, 0.58]	[−0.21, 0.66]

Confidence intervals are shown for selected coefficients from the supplementary models. * *p* < 0.05.

## Data Availability

The participant-level data are not publicly available because they contain potentially identifying information from human participants. Requests for access may be directed to the corresponding author and will be considered subject to institutional and ethical approvals.
